# Widespread Genomic Signatures of Natural Selection in Hominid Evolution

**DOI:** 10.1371/journal.pgen.1000471

**Published:** 2009-05-08

**Authors:** Graham McVicker, David Gordon, Colleen Davis, Phil Green

**Affiliations:** 1 Department of Genome Sciences, University of Washington, Seattle, Washington, United States of America; 2 Howard Hughes Medical Institute, University of Washington, Seattle, Washington, United States of America; University of Arizona, United States of America

## Abstract

Selection acting on genomic functional elements can be detected by its indirect effects on population diversity at linked neutral sites. To illuminate the selective forces that shaped hominid evolution, we analyzed the genomic distributions of human polymorphisms and sequence differences among five primate species relative to the locations of conserved sequence features. Neutral sequence diversity in human and ancestral hominid populations is substantially reduced near such features, resulting in a surprisingly large genome average diversity reduction due to selection of 19–26% on the autosomes and 12–40% on the X chromosome. The overall trends are broadly consistent with “background selection” or hitchhiking in ancestral populations acting to remove deleterious variants. Average selection is much stronger on exonic (both protein-coding and untranslated) conserved features than non-exonic features. Long term selection, rather than complex speciation scenarios, explains the large intragenomic variation in human/chimpanzee divergence. Our analyses reveal a dominant role for selection in shaping genomic diversity and divergence patterns, clarify hominid evolution, and provide a baseline for investigating specific selective events.

## Author Summary

Comparisons of the human and chimpanzee genomes have revealed that the frequency of sequence differences between these species varies dramatically across the genome. Previously proposed explanations for this variation include a large ancestral population, variable mutation rates, or a complex speciation scenario in which humans and chimpanzees initially separated but then rehybridyzed several million years later. We consider, here, an alternate possibility; the action of selection to remove less-fit functional variants from a population has significantly reduced the frequency of “neutral” sequence differences at nearby sites. We identified sequences that are likely to be subject to natural selection because they are highly conserved across placental mammals and showed that neutral differences among five primate species are greatly depleted near such sequences. Applying a theoretical evolutionary model, we found that selection has played a greater role in shaping hominid genome evolution than has been appreciated and provides a better explanation for patterns of sequence differences than other hypotheses.

## Introduction

The action of natural selection on genome sequences is most directly revealed by a deficit or excess of substitutions relative to the neutral rate, but detecting this requires sequences that have been diverging long enough to experience a high density of mutations [1]. An alternative approach, applicable over shorter evolutionary time periods, is to look for indirect effects of selection on neutral sequence variation [2],[3]. Directional selection reduces population diversity at linked neutral sites by eliminating chromosomes bearing a less fit variant from the population, an effect known as ‘hitchhiking’ in the case of positive selection [3] and ‘background selection’ in the case of negative or purifying selection [2],[4],[5]. The magnitude of the diversity reduction depends upon the density of selected sites, the amount of time during which selected variants segregate in the population prior to fixation or loss, and the rate at which recombination decouples neutral sites from selected variants [2],[4],[5]. In *Drosophila* a positive correlation between recombination rate and nucleotide diversity is well established and there is strong evidence for background selection or hitchhiking [2], [4], [6]–[10]. In hominid evolution, the roles of background selection and hitchhiking are less certain. Human diversity is positively correlated with recombination on a large scale [11]–[13] and negatively correlated with coding sequence density [14], consistent with a role for selection in recent human evolution. However, whole genome scans have identified relatively few regions with convincing evidence of positive selection [15],[16], an important role for background selection has generally been discounted [5],[17],[18], and it has been suggested that the association with recombination may reflect a mutagenic effect rather than selection [11],[17]. Consequently a clear picture of the importance and nature of selection in human evolution is still lacking.

**Figure 1:**
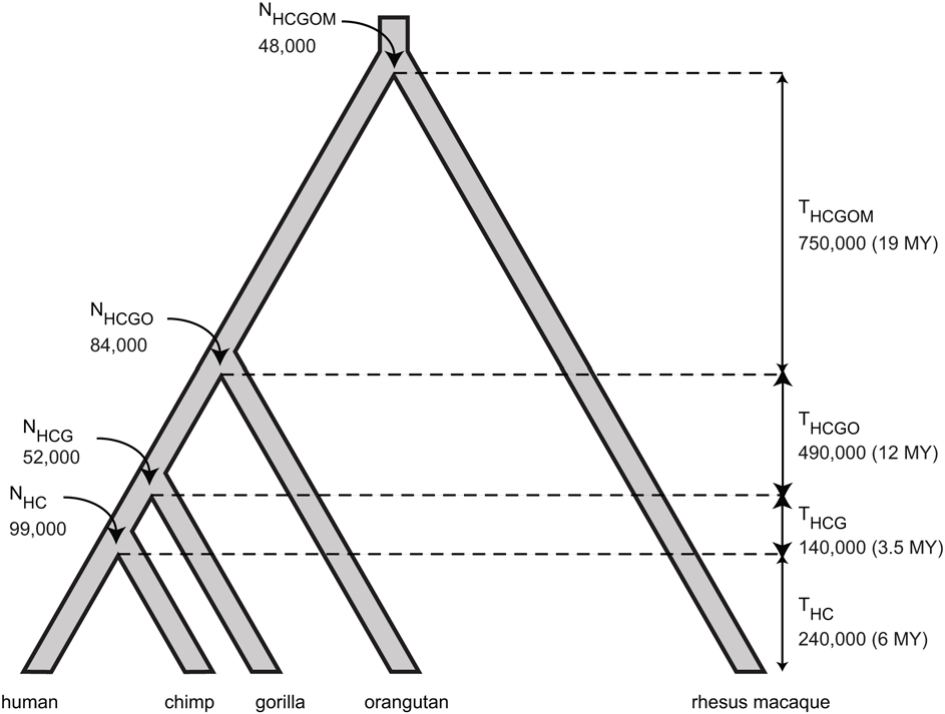
Species and populations analyzed. Ancestral effective population sizes, *N*, and interspeciation times in generations, *T*, were estimated by fitting a model of selection to five-primate sequence data (Table 1 contains all parameter estimates). Parameter values were calibrated by assuming human/chimpanzee speciation occurred 240,000 generations ago; a different calibration would multiply all values by a constant factor. The times between speciation in millions of years (MY) are shown in parentheses, assuming a constant generation time of 25 years. The old world monkey/great ape divergence time is older than suggested by the fossil record [82], but can potentially be explained by generation times that have increased during hominid evolution or a more recent human/chimpanzee speciation time than was used for calibration.

Here we conduct a broader and more systematic search for signatures of selection. We look more widely in hominid evolution, augmenting human polymorphism data [19],[20] with orthologous sequences for five primate species ([21] and our laboratory) (Figure 1). The latter sequences carry information about ancient population diversity, because some sequence differences between any two species represents polymorphic variation that existed in their common ancestral population [22].

## Results/Discussion

**Figure 2:**
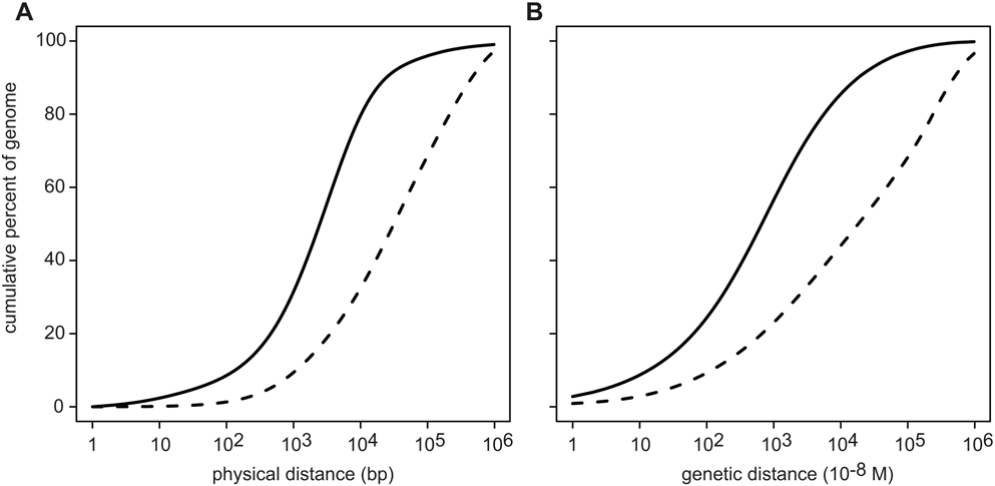
Most genomic bases are near a conserved segment. Plots show the percentage of the genome that is within a given distance of a conserved segment (solid curve) or protein coding sequence (broken curve). (A) Physical distances. (B) Genetic distances according to a fine-scale recombination map [25].

We used mammalian sequence conservation to identify two classes of genomic segments: “conserved” segments, which appear to be under long-term purifying selection, and “neutral” segments which are putatively free of selective constraint. Specifically, we employed a phylogenetic Hidden Markov Model (HMM) [23], which we extended to improve sensitivity by incorporating information from alignment gaps. We ran the HMM on a multiple alignment of placental mammals [24], but intentionally excluded data from the great apes (including human) and rhesus macaque to avoid biasing our subsequent analysis of sequence divergence in these species. Less than one-fourth of conserved bases identified by this approach are protein-coding, with the remainder largely of unknown function [23]; moreover, conserved segments are much more uniformly distributed in the genome than coding sequences, with most genomic bases surprisingly close to a conserved site (Figure 2). Thus it is desirable to take into account the detailed genomic distribution of all conserved sequences, and not just coding sequences, in investigating the effects of selection on diversity. Using sequence conservation rather than existing gene annotations has the advantage that it is unbiased by assumptions about which annotated features are functional.

**Figure 3:**
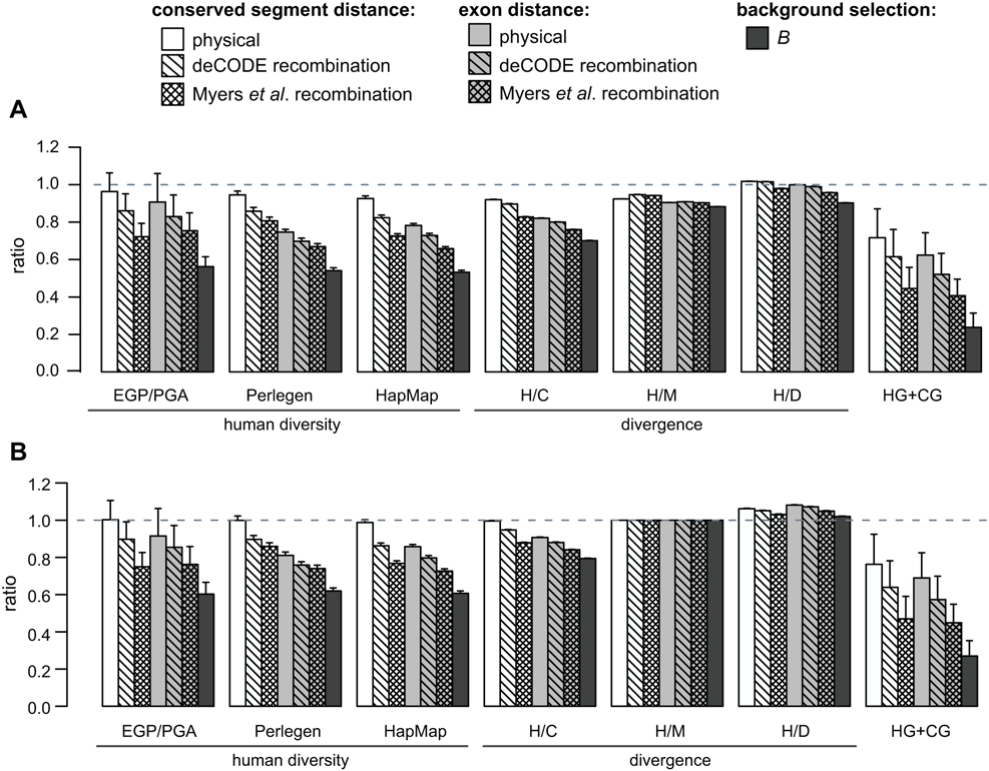
Human diversity, interspecies divergence and HG and CG sites are reduced near evolutionarily conserved segments. (A) Ratios calculated using the 10% of neutral sites which are nearest to and the 50% of neutral sites farthest away from conserved segments or exons. (B) The same ratios as (A) but normalized by human/macaque (H/M) divergence to account for mutation rate variation or undetected sites under purifying selection. The distance to the nearest conserved segment or exon was determined using four different measures: physical distance, pedigree-based recombination distance [26], polymorphism-based finescale recombination distance [25] and the background selection parameter, *B*. *B* (described in the main text) is not technically a distance measure but incorporates information about the recombination rate and local density of conserved segments. Autosomal human nucleotide diversity was calculated from gene-centric SeattleSNPs PGA/EGP [20], whole-genome Perlegen [19] data, and HapMap phase II data [67]. Divergence was estimated using autosomal human/chimp (H/C), human/macaque (H/M), or human/dog (H/D) genome sequence data. HG and CG sites (where human and gorilla or chimp and gorilla share a nucleotide that differs from the other three species) were calculated using a smaller set of 5-species autosomal data. Repetitive regions were omitted from the Perlegen and HapMap analyses; additional filtering steps are described in the methods. Whiskers are 95% confidence intervals.

**Figure 4:**
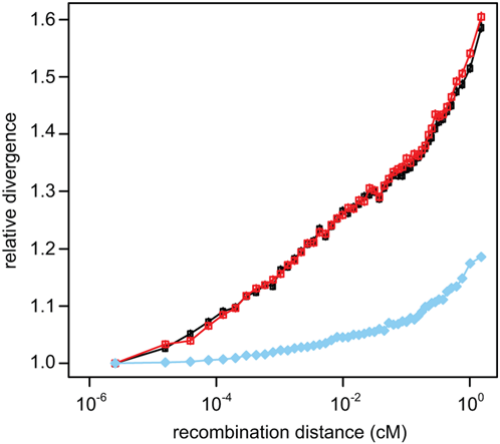
Neutral divergence increases with recombination distance from conserved exonic segments. Divergence in putatively neutral sites was calculated for the human branch (black circles), chimpanzee branch (red squares) and outgroup macaque branch (blue diamonds) and binned by finescale recombination distance from exonic conserved segments. Divergence is presented as relative to that of the first bin. Fifty bins of equal numbers of sites were used. Vertical lines are 95% confidence intervals.

We next compared levels of variation at putative neutral sites in the 10% of the genome nearest to conserved segments, to that in the 50% of the genome farthest from such segments, hypothesizing that selection should have a reduced effect on more distant regions. Human diversity and human/chimpanzee (H/C) divergence are indeed both substantially reduced near conserved segments, and using genetic instead of physical distance magnifies this effect (Figure 3). An even stronger reduction in neutral divergence and diversity is observed if distances are calculated with respect to annotated exons rather than conserved segments, suggesting that selection acting on exonic sequences has a greater effect on nearby diversity than selection on non-exonic conserved sequences. The effect is not limited to sites which are closest to exons; across the genome, H/C divergence exhibits a strong dependency on distance from conserved exonic segments (Figure 4, Table S1). Somewhat surprisingly, a fine-scale recombination map that incorporates ‘hotspot’ patterns [25] provides significantly better discrimination than a coarse pedigree-based map [26], even though many hotspots have moved in recent evolution [27],[28]. This suggests the finescale map may be more accurate than the pedigree map at smaller scales despite the hotspot movement.

The trends described above are consistent with selection at conserved segments acting to reduce diversity in both the human and human-chimpanzee ancestral populations. As a more sensitive indicator for the latter population, we also examined neutral sites where human and gorilla, or chimpanzee and gorilla, share one nucleotide and the other 3 primates share a different nucleotide (‘HG’ and ‘CG’ sites). At such positions, the human-chimpanzee coalescent predates the gorilla split [29] (see Figure S1) and so is very old. Since directional selection reduces time to coalescence at linked neutral sites, the density of HG and CG sites should be depleted near elements under selection, and this is indeed the case around conserved segments (Figure 3).

To control for the possibility that the lower diversity and divergence near conserved segments are due to the presence of unidentified sites under negative selection, or to a lower neutral mutation rate, we calculated human/macaque (H/M) and human/dog (H/D) divergence in the same bins. Only a small portion of divergence between distantly related species should reflect ancestral population diversity, so background selection or hitchhiking should have a minor effect on H/M divergence and a negligible effect on H/D divergence. There is a small reduction in both H/M and H/D divergence near conserved segments, suggesting that some of the trend is attributable to mutation rate variation or direct selection. However, normalizing by H/M divergence to cancel such effects does not change the overall trends (Figure 3) suggesting they are mainly due to indirect effects of selection. (Since some fraction of H/M divergence itself reflects ancestral diversity, normalizing in this way is an overcorrection, which is presumably why it reverses the trend for H/D divergence). We also confirmed that the same trends are seen separately for introns and for intergenic sequences upstream and downstream of transcripts (Figure S2).

Normalizing by H/M divergence would not correct for lineage-specific mutation rate variation. For example, if recombination is itself mutagenic [11],[13] and recombination rates have changed in primate evolution, normalizing by H/M divergence may fail to cancel recombination-induced mutation rate variation among hominids. However, we are unable to envision a plausible scenario along these lines that could explain the trends in Figure 3. In particular, changes in recombination would not explain the dependence on physical distance from exons.

**Figure 5:**
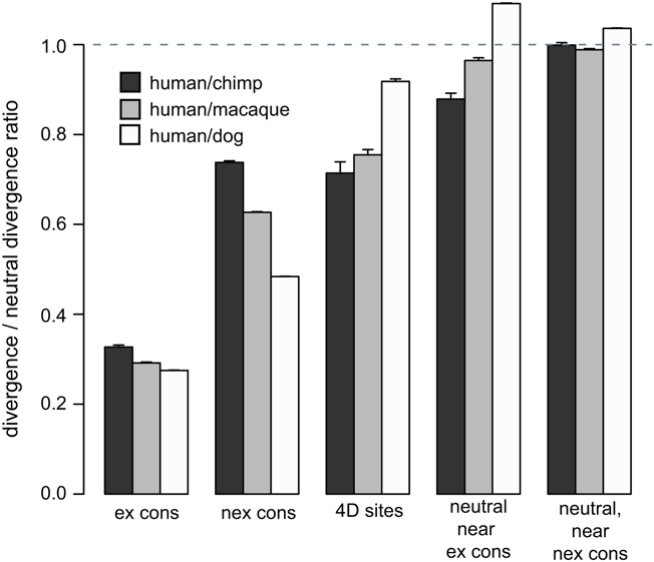
Divergence as a fraction of neutral divergence in conserved and neutral sites near conserved sites. We estimated human/chimp (H/C), human/macaque (H/M) and human/dog (H/D) divergence in exonic conserved segments (ex cons), non-exonic conserved segments (nex cons), fourfold degenerate (4D) sites (both neutral and conserved sites), and neutral segments within 100 bp of conserved segments using autosomal genomic alignments. These divergence estimates were then divided by the overall neutral divergence estimated from all autosomal neutral sites. The higher H/D divergence near conserved segments is likely an artefact of the Hidden Markov Model, which tends to terminate conserved segments at divergent bases (the dog sequence was used for conserved segment identification, but the human and macaque sequences were not). Whiskers are 95% confidence intervals.

We next examined the evolutionary rates within conserved sequences and putatively neutral sequences near conserved sequences, calculating divergence relative to the genome average at all putatively neutral sites (Figure 5). Relative divergence is much lower in exonic than non-exonic conserved segments, suggesting that selection is weaker on the non-exonic sites. The relative divergence in conserved segments decreases with evolutionary distance (*e.g.* relative divergence is lowest for the H/D comparison) consistent with weaker selection in the hominid lineage [30]–[32]. The opposite trend is observed for fourfold degenerate (4D) sites, and neutral sites near exonic conserved segments. In these cases relative divergence increases with evolutionary distance, which is consistent with background selection or hitchhiking, rather than direct selection. Divergence in 4D sites is substantially lower than the overall neutral rate even for the human-dog comparison, possibly because a subset of these sites are under direct selection. H/C and H/M divergence are only slightly lower in neutral sites near non-exonic conserved segments suggesting that background selection or hitchhiking in these regions is very weak.

The preceding analysis indicates a role for selection in shaping population diversity, but does not allow quantitative conclusions about selection strength. We therefore undertook a more detailed analysis, applying a theoretical model [5] of background selection to compute the expected reduction in nucleotide diversity at a neutral site due to purifying selection at other sites, as a function of recombination rates, selected site locations, deleterious mutation rate, and the distribution of selection strengths. We use a model of background selection rather than hitchhiking because it should provide a reasonable baseline estimate for the effects of selection, given that purifying selection is thought to be widespread (affecting most functional elements), while the relative importance of positive selection is still controversial. Because strength of selection in hominids may depend on the type of functional element [31] we distinguish exonic (protein-coding and UTR) from non-exonic selected sites, allowing them to have different mean selection strengths and deleterious mutation rates. From these calculations we obtain a background selection (*B*) value for each position in the genome. *B* indicates the expected fraction of neutral diversity that is present at a site, with values close to 0 representing near complete removal of diversity as a result of selection and values near 1 indicating little effect. We then represented the probability of the observed primate sequence alignment data as a function of species divergence times, mutation rates, ancestral effective population sizes, and *B*, and estimated all parameters by maximum likelihood (Table 1). Additionally, our model corrects for intragenomic mutation rate variation by allowing the mutation rate to vary with local H/D divergence.

**Table 1:** Model parameters estimated by maximum likelihood.

Param	Estimates (90%C.I.)		Description
	5SA	HCX	
***μ_I_***	7.0×10^−9^ (6.4×10^−9^, 7.4×10^−9^)	7.3×10^−9^ (4.1×10^−9^, 7.5×10^−9^)	Mutation rate for transitions (*I*) and transversions (*V*) (per-generation, per-filtered-site)
***μ_V_***	1.8×10^−9^ (1.7×10^−9^, 2.0×10^−9^)	2.0×10^−9^ (1.2×10^−9^, 2.1×10^−9^)	
***λ_I_***	2.4 (2.2, 2.6)	—	Double mutation rate multipliers for transitions (*I*) and transversions (*V*)
***λ_V_***	4.4 (3.5, 5.0)	—	
***u_ex_***	7.4×10^−8^ (6.0×10^−8^, 1.0×10^−7^)	1.6×10^−7^ (3.5×10^−8^, 1.8×10^−7^)	Haploid deleterious mutation rate for exonic (*ex*) and non-exonic (*nex*) conserved segments (per-site, per-generation, does not depend on filtering)
***u_nex_***	8.4×10^−10^ (2.3×10^−10^, 1.5×10^−9^)	0	
***t_ex_***	2.5×10^−3^ (2.5×10^−3^, 5.0×10^−3^)	1.3×10^−3^ (6.7×10^−4^, 3.3×10^−3^)	Mean selection coefficients for exonic (*ex*) and non-exonic (*nex*) conserved segments
***t_nex_***	1.0×10^−5^ (1.0×10^−5^, 1.0×10^−5^)	3.3×10^−5^ (1.3×10^−5^, 6.7×10^−2^)	
***T_hc_***	2.4×10^5^ (fixed)	2.4×10^5^ (fixed)	Interspeciation times (generations)
***T_hcg_***	1.4×10^5^ (1.1×10^5^, 1.7×10^5^)	—	
***T_hcgo_***	4.9×10^5^ (4.5×10^5^, 5.4×10^5^)	—	
***T_hcgom_***	7.5×10^5^ (6.9×10^5^, 8.3×10^5^)	—	
***N_hc_***	9.9×10^4^ (7.4×10^4^, 1.4×10^5^)	2.4×10^4^ (2.0×10^4^, 1.3×10^5^)	Neutral ancestral effective population sizes
***N_hcg_***	5.2×10^4^ (4.9×10^4^, 5.6×10^4^)	—	
***N_hcgo_***	8.4×10^4^ (7.1×10^4^, 9.7×10^4^)	—	
***N_hcgom_***	4.8×10^4^ (1.7×10^4^, 7.5×10^4^)	—	

Estimates are from a 5-species autosomal (5SA) dataset or human/chimpanzee chromosome X (HCX) dataset. The human/chimpanzee dataset was used for the X because of the small amount of 5-species data available for this chromosome. For both datasets the human/chimpanzee speciation time *T_hc_* was fixed at 240,000 generations, and the remaining *T*, *N* and *μ* parameters were scaled accordingly. The deleterious (*u*) and neutral mutation rate parameters (*μ*) are not directly comparable, because the neutral rate estimates reflect site filtering but the deleterious rate estimates do not. Confidence intervals were calculated from 100 iterations of a bootstrap procedure described in the Methods.

The model provides a good fit to the alignment data (Figure S3), indicating a strong dependence of divergence on predicted background selection in each ancestral population. Our speciation time and effective population size estimates are broadly consistent with previous analyses [29],[33],[34] (Table 1). The mean selection strength (*t*) estimate for autosomal exonic conserved segments is 0.0025, within the range of those from recent studies of human coding sequence polymorphisms [35],[36]. For non-exonic conserved sites, *t* is very low (0.00001); moreover fitting a reduced model that allows only for selection on conserved exonic segments gives essentially the same likelihood (Table S2) and parameter estimates (Table S3). This suggests that many non-exonic conserved segments are false-positives or are no longer under selection in hominids. The latter possibility accords with promoter region analyses that suggest weaker selection on regulatory elements in hominids than rodents, possibly because hominid effective population sizes are smaller [31]. If selection is weaker on non-exonic conserved elements in hominids then they should evolve more quickly in the human and chimpanzee lineages. A comparison of H/C, H/M and H/D divergence in these elements confirms that this is indeed the case (Figure 5).

Our estimate of the deleterious mutation rate at exonic selected sites (Table 1) substantially exceeds the per base mutation rate estimates from other studies [37],[38]. In part this excess may reflect background selection on deleterious mutations occurring outside our designated conserved segments, including mutations in other coding or exonic sites (only 63% of annotated coding bases meet our conservation threshold), and intronic mutations (including transposable element insertions) that affect splicing or polyadenylation. Widespread positive selection [39], fluctuating selection (which tends to amplify hitchhiking effects [40]), or biased gene conversion that increases the frequency of deleterious alleles [41],[42] may also contribute to the diversity reduction. We cannot at present distinguish among these possibilities, and consequently our *B* estimates should be interpreted as perhaps only partly reflecting background selection. A recent examination of human segregating sites by Hellman *et al.* found that both hitchhiking and background selection explain the relationship between diversity and recombination rate better than neutral models [43]. In their analysis the hitchhiking model gave a slightly better fit, but their results are not conclusive because their models are greatly simplified and in particular do not consider the distribution of conserved segments in the genome. We attempted to discriminate between background selection and hitchhiking models by examining allele frequency distributions in regions near or far from conserved segments (as in [8]). However, we were not able to find conclusive evidence that favored one model over the other (see Text S1, Table S4, and Figure S4). Both hitchhiking and background selection are likely to contribute to patterns of genomic diversity and future work would ideally take both forces into account [44].

**Figure 6:**
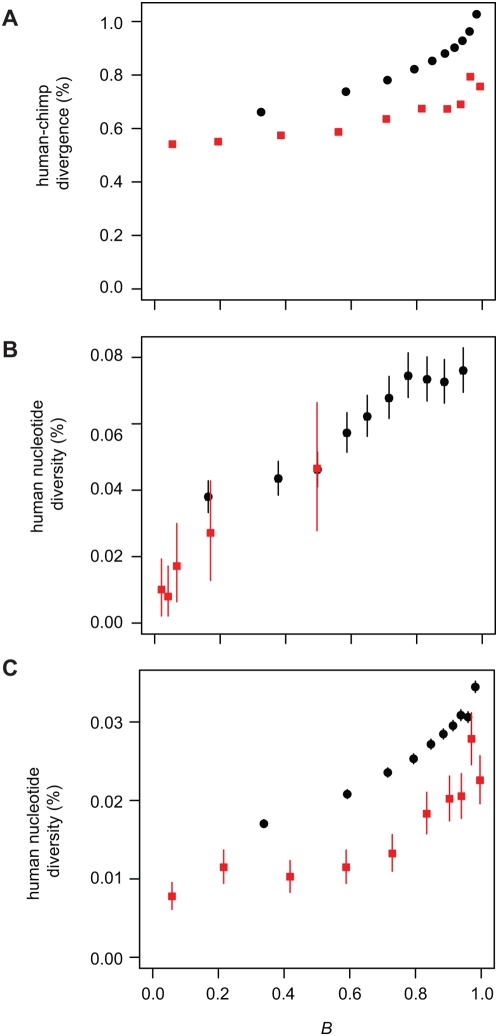
Whole-genome neutral divergence and diversity show strong dependence on the estimated strength of background selection. (A) Human/chimpanzee divergence from whole-genome alignments for autosomes (black circles) and chromosome X (red squares) versus *B* (the portion of neutral diversity expected to remain after accounting for background selection). (B) Human nucleotide diversity from Seattle SNPs PGA/EGP [20] data versus *B*. (C) Human nucleotide diversity from Perlegen [19] data. Estimated diversity is much lower in the Perlegen dataset because it subsamples common variants [19]. Vertical lines are 95% confidence intervals (not visible in (A) because they are smaller than the plotting symbols). Note that although human diversity shows a clear linear relationship to *B*, a fitted line would not pass through the origin as it should if the 5-species estimates are applicable to recent human evolution. This likely reflects the sharp decrease in human effective population size relative to ancestral primate populations, which is expected to reduce the efficiency of selection on weakly deleterious mutations due to increased genetic drift [31].

**Figure 7:**
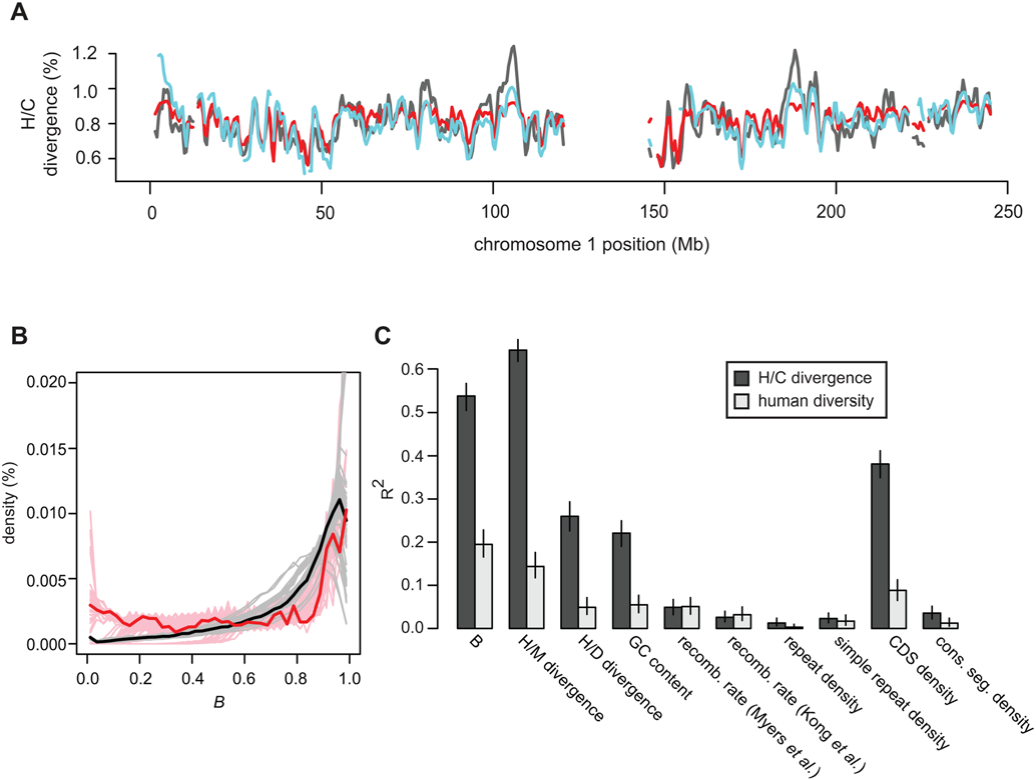
Selection can explain most large-scale regional variation in human/chimpanzee divergence and human diversity. (A) Observed (black line) and predicted H/C divergence across chromosome 1, from a background selection model that assumes a uniform mutation rate (red line) or a mutation rate that varies with local human/dog divergence (blue line). This plot was created with a 1 Mb sliding window with 0.5 Mb of overlap. (B) The distribution of estimated *B* values on autosomes (black line) and chromosome X (red line). Grey (autosomes) and pink (chromosome X) lines are distributions of *B* values from 100 bootstrap iterations. (C) Pairwise correlations (Spearman's rank squared) with regional human/chimpanzee (H/C) divergence and human diversity in non-overlapping 1 Mb windows across all autosomes. The same trends are observed across a wide range of window sizes (see Figure S6).Whiskers are 95% confidence intervals.

The mean autosomal *B* value predicted by our model is 0.74–0.81 (bootstrap 90% CI), indicating selection has reduced autosomal diversity by 19–26% on average during hominid evolution. Genome-wide H/C divergence shows a strong dependence on *B* (Figures 6A and 7), as does human diversity (Figure 6B,C) even after stratifying by local GC content or recombination rate (Figure S5). This genome-wide dependence is striking given that the model parameters were estimated using only a small set of genomic data (about 8.5 million filtered alignment columns for which 5-species data was available). To further quantify how well regional variation in neutral H/C divergence and human diversity can be explained by selection, we calculated correlations with divergence and diversity in non-overlapping genomic windows (Figures 7C and S6). Both *B* values and H/M divergence are well correlated with H/C divergence and human diversity. The correlation with H/M divergence is consistent with the action of selection because at least some variation in H/M divergence is attributable to selection in the ancestral population. H/D divergence exhibits a much weaker, but still substantial, correlation with H/C divergence. Since very little variation in neutral H/D divergence is likely to reflect selection in the ancestral population, this correlation is probably attributable to variation in the neutral mutation rate. H/C divergence is also well correlated with the density of protein coding sequences but not with the density of conserved segments (the majority of which are non-exonic). Thus, although selection on coding sequences appears to exert a strong influence on levels of neutral diversity, selection on non-exonic conserved segments may be too weak to have much effect in hominids.

We can also now interpret several puzzling observations in the literature. H/C divergence was observed to be elevated both in high-recombination and in A+T rich regions [45], which was attributed to the action of two different mutagenic forces. Both trends are at least partly explained by the association of divergence with *B*, since the effects of selection are weakest in regions where recombination is high or gene density is low, and A+T-rich regions tend to be gene-poor [46]. In comparison to *B*, factors previously proposed to influence local mutation rates such as recombination rate and GC content [17] are only weakly correlated with diversity and H/C divergence (Figure 7C and S6). This again suggests that selection, rather than mutation rate variation, is the principal reason for these associations.

Patterson *et al.* [21] proposed that the large variation in H/C divergence within the genome reflects relatively recent hybridization events following a much earlier split (a similar proposal was made earlier by Osada and Wu [47]). In contrast, Innan and Watanabe found no evidence supporting a model of gene flow following an initial speciation event [48] and Barton argued that much of the variation in divergence could instead be explained by a simple speciation model and a large ancestral effective population size [49]. Although a large ancestral population would give rise to genomic segments that differ widely in their H/C divergence and HG+CG site density [34], under a neutral model these segments would be scattered randomly throughout the genome. In contrast, we found that H/C divergence and HG+CG site density are preferentially depleted in the vicinity of conserved exonic sequences. This also contradicts the predictions of a complex speciation model that divergence should be lowest in intergenic regions [47]. Our results argue that much of the variation is instead attributable to the action of natural selection in a fairly large ancestral population (Figure 7).

An additional anomaly identified by Patterson *et al.* is the unexpectedly low divergence of the X chromosome relative to the autosomes. We analyzed the X chromosome using our likelihood model (Table 1) and found that, as with the autosomal analysis, the model provides a good fit to the data and reveals a strong dependence of ancestral population diversity on *B* (Figure 6). The estimated average diversity reduction for chromosome X is 12–41% (bootstrap 90% CI). At neutral sites not influenced by selection the estimated effective population size for the X is only 24% that of the autosomes (Table 1), however the large confidence intervals imply that this is not significantly different from the 75% expectation of random mating models. Because of the uncertainty in our chromosome X parameter estimates we cannot determine whether the low H/C divergence across the chromosome can be explained by selection. The future availability of complete genome sequences from gorilla and orangutan should enable a more precise comparison of chromosome X and the autosomes.

In a recent study of human diversity (published while this manuscript was under review) Cai *et al.* estimated hitchhiking or background selection has reduced neutral diversity by 6% genome-wide (11% in gene-rich regions) [50]. Their estimate is substantially lower than our own (19–26% for autosomes), but the discrepancy can potentially be explained by several aspects of their analysis. They exclude all sites near genes (within 5 kb of transcript start and ends and within 1 kb of any exon); since about 11% of the genome is within 1 kb of an exon this omits a large fraction of the sites that are the most influenced by selection. In addition, their analysis uses very large windows (400 kb) which will tend to dilute some of the effects of selection. Finally, they normalize human diversity by H/C divergence as a correction for mutation rate variaton. This normalization is overly conservative because as we have shown here, a substantial fraction of H/C divergence is itself affected by selection.

In summary, our analyses reveal a dominant role for selection in shaping genomic patterns of diversity and divergence, and appear to resolve several controversies regarding hominid evolution. Our results have several implications for studies that involve human diversity or H/C divergence. Findings of reduced H/C divergence in some regions may reflect the indirect effects of selection at nearby sites, rather than direct selection or reduced mutation rates. For example, the lower H/C divergence in short introns [51] might reflect selection on nearby exons. In addition, estimates of the effective population size or neutral mutation rate should be based on regions that are distant from selected sites. The *B* values computed by our model should provide a useful baseline for future studies, allowing regions to be stratified by their predicted levels of neutral diversity or divergence. Loci that depart significantly from our diversity predictions warrant more detailed investigation because they may have undergone unusually strong selective or mutagenic events.

## Methods

### Genome Sequences and Annotation

Genome sequences for the human [46] (version hg18), chimpanzee [45] (version panTro2), and rhesus macaque [52] (version rheMac2) genomes, and human genome annotation files were obtained from the University of California at Santa Cruz Genomic Informatics (UCSC) web site [53]. Human protein-coding sequences and exons were identified using UCSC ‘known gene’ files [54] (downloaded Sept. 2007). Repetitive regions were identified using the UCSC lower-case markup (which is based on RepeatMasker [55] and Tandem Repeats Finder [56] analysis). Simple repeats identified by Tandem Repeats Finder were also downloaded from the UCSC simpleRepeats track so that they could be used independently.

### Recombination Rates

Files indicating map distance per nucleotide for deCODE [26] and Myers *et al.* [25] recombination maps were downloaded Feb 2007 (we used snpRecombRateHapmap files for the Myers *et al.* map), and transferred from hg17 using the UCSC liftOver tool. X chromosome values were multiplied by 2/3 to correct for non-recombination in males. Chromosome regions missing from the recombination maps were ignored for most analyses; however for use in calculating background selection values, we assigned each base in missing regions a recombination rate equal to that of the nearest defined base (for terminal regions of chromosomes) or the mean of the nearest defined bases from each side (for internal regions).

### Human–Chimp–Macaque Whole-Genome Alignments

We downloaded ‘chained and netted’ pairwise whole genome alignments from UCSC [57]–[59] for human (hg18), chimp (panTro2) and macaque (rheMac2). We converted these ‘best’ alignments to be best-reciprocal by splitting alignment blocks to omit portions that were non-reciprocal between forward (*e.g.* hg18 vs. panTro2) and reverse (*e.g.* panTro2 vs. hg18) alignments. Next, blocks aligning parts of non-orthologous chromosomes or unassigned to a chromosome region were discarded. Human and macaque chromosome regions were considered orthologous if their pairing was consistent with the synteny map of Rogers *et al.* [60] and for the sex chromosomes, only X to X and Y to Y alignment blocks were kept, in accordance with the synteny map of Murphy *et al.* [61].

We filtered out putative copy number variants and segmental duplications since these are likely to be enriched for non-orthologous alignments. Alignment blocks were omitted if more than 50% of a block overlapped regions identified as having an excessive depth of shotgun sequence reads (WSSD regions). WSSD features generated from Celera, Venter, and Watson human genome sequences as well as chimpanzee and orangutan sequencing projects were combined in order to create the set used for filtering [62],[63]. Additionally, human-chimp alignment blocks were excluded if the chimp sequence overlapped WSSD features identified by aligning chimp reads to the chimp genome, and human-macaque alignment blocks were excluded if they overlapped WSSD features identified by aligning macaque reads to the macaque genome [63].

We then grouped remaining alignment blocks into ‘chains’. Blocks were chained when their chromosomal ordering was consistent for both species. We eliminated chains with fewer than 250 kb in the human-chimp alignment, or 50 kb in the human-macaque alignment. We further excluded blocks with lengths less than 2 kb from both alignments.

The remaining pairwise human-chimp and human-macaque alignments were then used to define a three-species alignment. We applied a set of site filters to individual alignment columns. We used only sequence with high-confidence base calls, requiring that each site was flanked by five sites with minimum quality scores 25 (in both chimp and macaque), and that the site itself had a quality score of at least 40. We ignored columns in the alignment that included gap characters, were adjacent to mismatches, gaps or undefined bases, or that overlapped a CpG dinucleotide in any of the three species. We also imposed a ‘symmetry’ filter to eliminate potential non-orthologous alignments by using macaque as an outgroup to assign (where possible) human-chimpanzee sequence differences to either the human or chimp branch, and eliminating regions in which more than 16 out of 20 successive substitutions were on the same branch.

### Human–Dog Whole-Genome Alignments

We downloaded pairwise human (hg18) and dog (canFam2) [64] alignments, converted these to best-reciprocal alignments as described above, and discarded blocks of length<100 bp and blocks that were unassigned to a chromosome region. Chains of blocks that had a combined length of <5000 bp were then discarded in a subsequent pass.

Regional human/dog divergence was estimated by counting alignment columns in putative neutral sites (defined below) in 1 Mb sliding windows that were advanced by 1 bp at a time. Only transversion substitutions were used in divergence calculations because they gave better correlations to human/macaque divergence than transition substitutions or Kimura-corrected divergence (presumably because many sites have multiple transition substitutions). Sites were excluded from the analysis if they were in a potential CpG context (following a C or preceding a G) or if they were adjacent to a gap or N. Windows were discarded if they contained fewer than 200,000 sites post-filtering.

For comparison of H/C, H/M and H/D divergence (Figures 3, 7C, S2 and S6) we constructed the implied human-dog-macaque alignment and applied the same filtering criteria used to calculate regional H/D divergence, again using only transversion substitutions. The same alignment was used to calculate levels of divergence in several site classes relative to the neutral rate (Figure 5). In this case, less stringent filtering was applied (sites in a potential CpG context were still excluded), and all four species were required to have an aligned base at each site.

### 5-Species Primate Data

We downloaded HCGOM alignments utilized in [21] from http://genepath.med.harvard.edu/~reich, and extracted columns identified as used in that study (these all have at least one nucleotide difference among species). We eliminated columns that overlapped a CpG dinucleotide in any species or that were not flanked by invariant columns (*i.e.* columns for which all species shared the same nucleotide). In addition, we identified all invariant columns, and selected at random a subset of these equivalent to the number reported in [21].

We augmented these data with a smaller dataset generated in our own laboratory as follows. We chose primer pairs to amplify 591 human genome loci of size 1 to 2 kb spaced roughly every 5 Mb on the autosomes and 2.5 Mb on the X. Primers were chosen to avoid repetitive regions, positions where human, chimp, or macaque differed, and the dinucleotide CpG, and trinucleotides ACA, or TGT which we have found to have higher than average mutability in primate sequences (data not shown). DNA samples from a male chimpanzee (*Pan troglodytes*), a male bonobo (*Pan paniscus*), a female gorilla (*Gorilla gorilla*), a female Sumatran orangutan (*Pongo pygmaeus*) and a female rhesus monkey (*Macaca mulatta*), purchased from the Coriell Institute (Camden, NJ), were PCR amplified and sequenced in both directions, using standard protocols and an ABI Prism 3100 Genetic Analyzer. After basecalling with *phred* [65],[66], we searched each read against the human genome and eliminated reads for which the best match was to a non-target location. The remaining 4759 reads were aligned to the human sequence using a banded Smith-Waterman algorithm (cross_match; www.phrap.org). Analyzed data were required to pass quality and alignment filters similar to those described [21]. Traces have been deposited in the NCBI trace archive (http://www.ncbi.nlm.nih.gov/Traces).

A total of 8.5 million alignment columns passing these filters in the combined 5-species dataset were analyzed.

### Human Diversity Data

Human single nucleotide polymorphism (SNP) data was obtained from Perlegen Sciences [19], HapMap phase II (non-redundant October 2008 update) [67], the SeattleSNPs NHLBI Program for Genomic Applications (PGA) [68] and the NIEHS Environmental Genome Project (EGP) [20],[69] (downloaded July 2008).

To estimate nucleotide diversity we averaged combined-population heterozygosity for all di-allelic polymorphisms in scanned regions (assuming heterozygosity of 0 for monomorphic sites). For the HapMap data the CEU panel was used instead of combined-population heterozygosity. As with divergence calculations, we required the presence of an aligned macaque base (necessary for normalization) and excluded sites in a CpG context, sites with poor quality scores, and sites adjacent to a gap or N in the human/macaque alignment. For both HapMap and Perlegen datasets we omitted sites that fell within annotated repeats.

Diversity for a given class of genomic sites (*e.g.* putative neutral sites having a specified background selection value) was estimated by summing the estimated heterozygosities (computed from observed allele frequencies in the samples) of SNPs in that class and dividing by the total number of scanned sites in the class.

To avoid ascertainment bias in the Perlegen data we only used class A SNPs, which had been identified using array-based resequencing [19]. We converted the NCBI34/hg16 coordinates of the Perlegen SNPs to NCBI36/hg18 coordinates using the UCSC-annotated positions of the associated dbSNP identifiers. The following sites were considered unscanned in order to correct for biases in Perlegen array-based detection: sites within 25 bases of an annotated repetitive region; sites with <6 or >13 G or C nucleotides among the 24 bases (12 to each side) flanking the site (our unpublished analyses indicate that class A SNPs are strongly depleted at such positions); regions >100 kb that completely lacked class A SNPs; and regions present in the NCBI36 assembly but not the NCBI34 assembly (as identified by mapping non-overlapping 1 kb segments from NCBI36 to NCBI34 using liftOver).

To address the possibility that Perlegen or HapMap SNP ascertainment strategies could bias our estimates of human diversity [70], we employed an ascertainment correction that takes into account the size of the discovery sample [71]. The discovery sample size of the Perlegen data is 20–50 chromosomes (see supplemental data for [19]). We were unable to obtain per-SNP discovery sample sizes so we calculated corrected nucleotide diversity values assuming uniform discovery sample sizes of either 20 or 50. Note that this ascertainment correction does not account for failure of the array technology to identify SNPs during the discovery process, but it should not bias our Figure 3 analyses (which compare regions near and far from conserved segments) provided discovery sample size and technology failures are not themselves biased with respect to distance from conserved segments. As expected, our ascertainment corrected nucleotide diversity estimates are higher than our uncorrected estimates, but our diversity ratios from regions near-to and far-from conserved sites are essentially unchanged (Table S5). Moreover, consistent with this expectation, we obtained similar results from HapMap phase II data, which used different ascertainment methodologies, and from SeattleSNPs EGP/PGA data derived from complete resequencing.

### Gcons Conservation Model

We implemented a program, gcons, to identify evolutionarily conserved segments from aligned genomic sequences. Gcons extends the two-state phylogenetic Hidden Markov Model (phylo-HMM) approach used by phastCons [23] by incorporating alignment gap information. We define separate substitution models for nucleotide and gap evolution, and estimate substitution probability matrices on each branch of the phylogenetic tree without assuming a common rate matrix (phastCons uses a single rate matrix). Our probability matrices are constrained to be strand-symmetric (*e.g.* A→G substitutions must occur at the same rate as complementary T→C substitutions) but may be non-reversible. Our gap substitution model is a simple site-independent deletion model with three symbols representing defined bases (*b*), sites in short gaps of length≤10 bp (*-*), and sites in long gaps or unaligned regions (*D*). Because we consider only ‘ancient’ sites present in the root and assume that orthologous nucleotides are aligned, it is unnecessary to model insertions. Thus the only non-zero substitution rates are *b*→*-*, *b*→*D*, and *-*→*D*. In high coverage genomes, the absence of long gaps is indicative of functional constraint [72], but in low-coverage genomes, long gaps may simply represent coverage gaps and are therefore less informative [24]. Because our model uses separate long and short gap symbols and allows rates to vary on different branches, it can be applied effectively to a mixture of high and low sequence coverage genomes.

From a set of alignment columns we obtain maximum likelihood estimates of the substitution probability matrices on each branch of a phylogenetic tree using an EM algorithm [73]. For our purposes, it is sufficient to estimate substitution probabilities directly, rather than the underlying substitution rate matrices and branch lengths.

We downloaded a multiple alignment of 28 vertebrate genomes from UCSC in August 2007 [24] and extracted from this the alignment of placental mammal species. To avoid biasing our primate sequence analyses we excluded chimpanzee and rhesus macaque sequences from the alignments, leaving a total of 15 sequences plus human and we treated the human sequence as missing data for the likelihood calculations described below. For these sequences we assume the following fixed phylogenetic tree topology obtained from UCSC (http://hgdownload.cse.ucsc.edu/goldenPath/hg18/multiz28way/28way.nh): *((((hg18,otoGar1),tupBel1),(((rn4,mm8),cavPor2),oryCun1)),((sorAra1,eriEur1),(((canFam2,felCat3),equCab1),bosTau3)))*.

We restrict our analysis to ‘ancient’ sites defined as those present in at least one species on either side of the internal node *((((hg18,otoGar1),tupBel1),(((rn4,mm8),cavPor2),oryCun1)),((sorAra1,eriEur1),(((canFam2,felCat3),equCab1),bosTau3)))*. This node is used instead of the root because the three species on one side of the root (armadillo, elephant, tenrec) have low-coverage (2×) assemblies with many gaps.

We estimate neutral substitution probabilities using multiple alignment columns from ancient repeats. Repeats were identified using the lower-case markup in the UCSC human hg18 sequence; to allow for repeat alignment ambiguity we excluded the 5 bp at each end of the repeat. Ancient sites that fulfilled these criteria were considered to be ancient repeats. Similarly, we used first and second codon positions in annotated coding sequences to estimate the conserved region substitution probabilities. Alignments from odd-numbered autosomes were used as a ‘training set’ input for the EM algorithm, and data from even-numbered autosomes were used as a ‘test’ set. Substitution probabilities for the X chromosome were estimated using the full set of alignment data (*i.e.* no test set was held out).

To approximate flanking nucleotide context effects on substitution rates [74] we categorized sites by their inferred ancestral context and trained separate models for each category. Specifically, for each ‘ancient’ alignment column we designated an ancestral nucleotide by choosing the nucleotide with the highest posterior root probability, as calculated using our initial (context-free) neutral evolutionary model. We then grouped alignment columns into categories based upon their ancestral purine and pyrimidine contexts because these contexts have previously been shown to capture a substantial proportion of mutation rate variation [74]. The four possible context categories are RRR, RRY, YRR, YRY, where the center symbol is the ancestral state at the site of interest and R and Y denote purine and pyrimidine, respectively (note that reverse-complement pairs of contexts, *e.g.* RRY and YYR, are equivalent by virtue of the strand symmetry condition on our substitution matrices). After grouping columns by their ancestral contexts we trained separate conserved and neutral evolutionary models for each possible context as described above, retaining the initial context-free model for sites where one of the flanking ancestral states is unknown.

We then computed a conserved/neutral log-likelihood ratio (LLR) for each ancient site in the human genome using these models. The LLR for non-ancient and unaligned sites was taken as the log of the rate of occurrence of such sites in conserved regions (first and second codon positions) divided by the rate of such sites in neutral regions (ancient repeats). To avoid biasing our primate sequence analyses, human sites were treated as missing data in LLR calculations.

The sum of the nucleotide substitution and gap LLRs at each site may be interpreted as the log of the ratio of the emission probabilities of the corresponding alignment column by a Hidden Markov Model having two states, ‘conserved’ and ‘neutral’. We assigned state transition probabilities of 1/7 (*conserved*→*neutral*) and 0.0075 (*neutral*→*conserved*), implying an expected conserved segment length of 7 bp and an expected conserved portion of the genome of 5%, and computed a score, *S*, for each site which is related to the posterior probability, *PP*, of being in the conserved state by *PP* = *e^s^*/(*e^s^*+*1*).

To identify potentially incorrect portions of the multiple alignment we used a similar procedure, defining an HMM with a neutral and a ‘high substitution’ state. Emission probabilities for the neutral state used the context-free substitution probability matrices from ancient regions, whereas those matrices raised to the 5^th^ power defined emission probabilities for the high substitution state. State transition probabilities were chosen such that high substitution segments were expected to be of length 25 bp and span 10% of the genome. We then computed scores as above, and defined contiguous regions with scores greater than 0.0 (posterior probability 0.5) as high substitution segments; these comprise 8% of aligned ancient repeats, 2% of aligned intergenic bases and 0.2% of aligned first and second codon positions. These segments likely reflect misalignments, and we excluded them before re-extracting alignment columns and re-performing the training of the conserved and neutral models described above (*i.e.* the columns were omitted for training, but retained in other analyses).

### Conserved Segment and Neutral Site Identification

We defined *conserved segments* to be contiguous sets of bases in the human genome having gcons score≥10; these are the bases with the strongest evidence for being under purifying selection. Note however that because the gcons model is designed to detect segments of a given minimal length rather than individual conserved bases, some bases within a conserved segment may be under little or no selection pressure (*e.g.* synonymous bases within coding exons), and short evolutionarily constrained segments may have low gcons scores. Approximately 39% of annotated exonic bases and 4.3% of non-exonic bases meet our gcons score threshold. We classified conserved segments as ‘exonic’ if they contain any annotated exonic base, and as ‘non-exonic’ otherwise. Exonic and non-exonic conserved segments comprise 1.1% and 4.2% of all genomic bases respectively.

Except where indicated, all analyses use *putative neutral sites*, which are required to be ≥10 bases away from any annotated exon, have gcons score<−10, and to pass the additional filters indicated above.

Gcons score thresholds of −10 and 10 were chosen to designate neutral and conserved sites because we found that they provide good separation between putative functional sites (*e.g.* known protein coding sequences) and putative non-functional sites (*e.g.* ancient repeats) (data not shown).

Following filtering, our primary datasets consisted of the following numbers of neutral sites: 1.2 billion autosomal and 48 million X chromosome human/chimp/macaque alignment columns; 550 million autosomal human/macaque/dog autosomal alignment columns; 5.3 million 5-species autosomal alignment columns; 6.5 million autosomal and 0.23 million X chromosome SeattleSNPs PGA and EGP scanned sites. Additional filtering steps were performed for the maximum likelihood analysis described below (regional H/D divergence was required to be defined, and some column types *e.g.* HGO, were not used). In total, 4.7 million alignment columns were retained for the 5-species autosomal dataset and 27 million alignment columns were retained for the chromosome X human/chimp dataset.

### Background Selection

We applied the model of Nordborg *et al.* [5] and Hudson and Kaplan [4], which estimates the expected reduction in nucleotide diversity at a neutral site due to purifying selection at other sites as a function of deleterious mutation rate, selection strength, and recombination rate, under the assumptions that selection acts multiplicatively over loci and is strong enough that allele frequencies of deleterious mutations remain low (so that homozygotes for the deleterious allele may be ignored). Specifically, the background selection coefficient *B* = *B* ^(ν)^ at a neutral site ν is given by
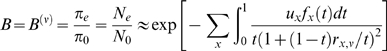
where

*π_e_* is the expected diversity at ν.
*π_0_* is the expected neutral diversity in the absence of background selection.

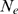
 is the effective population size at ν.

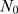
 is the effective population size in the absence of background selection.
*x* ranges over selected sites.
*u_x_* is the (haploid) deleterious mutation rate per generation at *x*.
*r_x_*_,ν_ is the recombination rate per generation between ν and *x*.
*t* is the strength of selection on heterozygotes: (1−*t*) represents the fitness of an individual heterozygous for a deleterious allele at *x*, relative to an individual homozygous for the normal allele. For a site on the X chromosome, which spends 1/3 of its time in males and 2/3 in females, *t* = *t_m_*/3+2 *t_f_*/3, where *t_m_*, *t_f_* are the selection strengths in hemizygous males and heterozygous females carrying the deleterious allele.
*f_x_*(*t*) specifies a probability density for alleles of varying selection strengths at *x*.

We distinguish two different classes of selected sites *x*, exonic and non-exonic, and allow them to have different *u* and *f*. Accordingly *B* may be expressed as a product *B* = *B_ex_ B_nex_* where
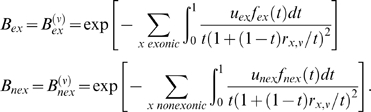



Using the above formulae we computed *B_ex_* and *B_nex_* values for each site ν in the human genome, for various selection densities *f*, and *u_x_* fixed (initially) to a value of 1.2×10^−8^. The selected sites *x* are taken to be the bases in exonic or non-exonic conserved segments as defined above, and *r_x_*_,ν_ is taken to be the recombination map distance between ν and *x* (this slightly overestimates the actual recombination rate). For *f* we use truncated exponential distributions: *f*(*t*) = 0 for *t*>1 and *t*<10^−5^, and *f*(*t*) = *C* e^−*ct*^ for 10^−5^≤*t*≤1 where *c* and *C* are constants. We considered *f* having mean values of the form *a*10*^b^*, (or (4/3) *a*10*^b^* in the case of the X chromosome) where *a* = 5.0, 2.5, or 1.0, and *b* = −2, −3, −4, or −5. As alternative possibilities for *f* we also considered point distributions, and truncated gamma distributions with shape parameters 0.25, 0.75 and 2.0 (using the same grid of mean values). The gamma distribution with shape parameter 0.75 gave a slightly higher likelihood for the 5-species autosomal dataset, but not significantly so given the additional degree of freedom (Table S2). For the human/chimp chromosome X dataset a point distribution gave a slightly better likelihood (Table S2), but for consistency with the autosomal analysis we use the exponential distribution results. Classifying conserved segments as coding or non-coding rather than exonic or non-exonic, or using the deCODE [26] instead of a finescale recombination map [25], gave somewhat lower likelihoods in our preliminary analyses (data not shown).

To accelerate calculations of *B_ex_* and *B_nex_* we employed several approximations. We constructed a lookup table giving, for a range of values of *r* and the length of the conserved segment, values of the integral (evaluated numerically) over *f*. Integrals were then estimated by performing bilinear interpolation between the nearest values stored in the table. Summations over *x* were done segment-by-segment, approximating the sum over the segment by a continuous integral. To make this approximation more accurate, segments were broken at points where the recombination map rate per nucleotide changed. The summations over segments were then performed by starting with segments nearest to ν and moving progressively farther away on the chromosome, calculating at each step the maximum possible remainder of the summation for the entire chromosome, and stopping the summation when this maximum remainder fell below a target value (0.001). Values for the first and second derivatives of the *B*'s (with respect to the position of ν) were computed by summing the term-by-term derivatives. Finally, we carried out summations only for a subset of ν's on the chromosome, with *B* values for other sites estimated by quadratic interpolation using the derivatives.

Our *B* value estimates are available for download from http://www.phrap.org.

### Likelihood Model

We model the probability of the observed 5-species alignment data as a function of species divergence times, ancestral effective population sizes, and background selection on exonic and non-exonic conserved segments, in order to estimate these parameters by maximum likelihood. Our model allows for the fact that the gene tree varies along the sequence, such that at a given site any two of human, chimp, or gorilla may share the most recent common ancestor (Figure S1). Following [21] we ignore alignment columns having more than two distinct nucleotides (implying two or more mutation events at the same position), and we label those with exactly two distinct nucleotides by indicating which species share the same nucleotide; thus an HG (or equivalently COM) column, or ‘site’, is one such that human (H) and gorilla (G) share one nucleotide, while chimp (C), orang (O) and macaque (M) share a different nucleotide. We ignore most site types such as HGO which represent obligate double mutation events, however we use HO and CO counts to help estimate rates of double mutation (described below). We assume each site involves a mutational change along at most two branches of the gene tree at that position; because all branches are short, multiple events are rare.

The probabilities that the sequences at the beginning and end of branch *i* differ by a transition (*I*) or transversion (*V*) substitution are given by Kimura's formulae [75]:
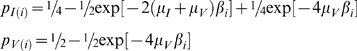
where *μ_I_* and *μ_V_* are the per-generation per-nucleotide transition and transversion mutation rates (so that the combined mutation rate is *μ* = *μ_I_*+2*μ_v_*), and β_i_ is the branch length (in generations). The probability of an observed column of type *k* is then
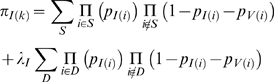
if the column has two distinct nucleotides differing by a transition;
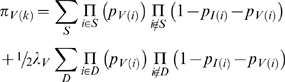
if the column has two distinct nucleotides differing by a transversion; and
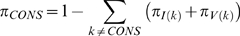
if the column is invariant.

Here *S* and *D* denote sets of branches that can give rise to the observed column type via a single or double substitution, respectively. Distinct alignment columns are treated as independent observations. The parameters λ_I_ and λ_V_ are used to scale the rates of double substitution events, which are higher than predicted by the site-independent Kimura substitution model because of mutational hotspots and flanking nucleotide contexts. Patterson *et al.* [21] observed that it is particularly important to take recurrent mutation into account for HG and CG columns, a significant fraction of which are the result of substitutions on multiple branches. We calculated the expected number of sites that are due to recurrent substitutions under our fitted model and compared the results to those from Patterson *et al.* Our estimates are in close agreement for the column types that are most frequently due to double substitution (HC, HG, and CG) (Table S6). We estimate lower rates of double substitution for some of the other column types, but since only a small fraction of these are due to double substitutions, differences in these rates should not affect our overall results.

To illustrate these issues consider the alignment column GAGAA, where human and gorilla both have a G nucleotide and the other three species have an A. This column could be the result of a single A→G transition substitution on the HG branch (assuming a gene tree that differs from the species tree) but could also be due to A→G transitions on both H and G branches, or an A→G transition on the HCG branch and a back substitution (G→A) on the C branch. In this case *S* is (*HG*), and *D* is either (*H*,*G*) or (*HCG*,*C*).

Expected branch lengths β [21],[29],[76] for each site type are given by:
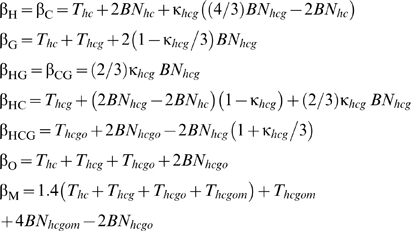
where 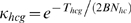 is the probability that the human-chimpanzee coalescent predates the gorilla speciation, the 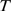
'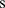 represent inter-speciation intervals (measured in generations), the 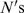 represent ancestral effective population sizes (corresponding to 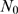 in the formula for *B* in the *Background selection* section above, and as depicted in Figure 1), and *B* = *B_ex_B_nex_* is the background selection value. The factor 1.4 in the β_M_ formula corrects for the estimated mutation rate excess in old world monkeys relative to hominids [77]. Note that in contrast to the other parameters, *B* depends on the sequence position. *B* also depends on the choice of recombination map, and on *u_ex_*, *u_nex_*, *f_ex_*, and *f_nex_*.

We assume the human-estimated *B* values apply to the orthologous bases in the other species, which is only approximately true because local recombination rates vary over time [27],[28]. Selection strengths may also vary, and even if they do not, differences in effective population size imply that deleterious mutations eliminated by selection in some populations may become fixed in others.

For the 3-species (human/chimpanzee/macaque whole genome alignment) analyses we developed a similar model, but ignoring the macaque branch and using 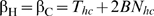.

To accelerate the probability calculations, we binned sites by their *B* values and column types. The log-probability of the data is then
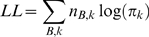
where *n_B,k_* is the number of filtered columns of type *k* in bin *B*, and *π_k_* is the probability associated to column type *k* as given above.

For each maximum likelihood analysis, the distribution functions *f_ex_* and *f_nex_* are held fixed to compute *B* across the genome for a particular *u_x_*, and estimates for the remaining parameters are obtained by searching the likelihood surface with the GNU Scientific Library's [78] implementation of the Broyden-Fletcher-Goldfarb-Shanno (BFGS) quasi-Newton method [79] (with slight modifications to prevent stalling at ridges), using analytically computed first partial derivatives. We varied *u_ex_* and *u_nex_* by rescaling *B* values (computed initially with a fixed *u*) as follows (where *i* denotes *ex* or *nex*, and 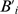 and 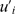 denote the updated values):
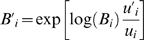



Because *μ* is confounded with the 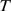 and 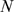 parameters, a calibration is required to infer individual parameter values; we fix 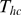 to 240,000 generations (assuming a species divergence time of 6 MYA and a 25 year generation time), and adjust the other 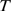 and 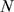 values proportionately. Note also that *μ* is distinct from *u_x_* (deleterious mutation rate per selected site, for calculating *B*): in particular *μ* reflects alignment filtering whereas *u_x_* does not, and the estimate of *u_x_* is influenced by background selection arising from deleterious mutations at sites outside the identified conserved segments.

Regional variation in neutral substitution rates [80] has the potential to bias our parameter estimates. In particular, a higher average neutral substitution rate in regions which are distant from conserved segments (potentially due to a mutational effect associated with recombination [11],[17] or insertions and deletions [81]), could be misinterpreted as evidence for selection in the ancestral population. To incorporate regional substitution rate variation into our model, we allowed mutation rates to depend upon regional human/dog divergence. Alignment column counts used for maximum likelihood estimation were binned by the regional human/dog divergence *D* in addition to *B_ex_* and *B_nex_*. Rather than estimating the transition and transversion mutation rate parameters (*μ_I_* and *μ_V_*) directly we instead estimate parameters *μ_A_* and *μ_B_* and define the transition and transversion rates in each bin as *μ_I_* = *μ_A_D* and *μ_V_* = *μ_B_D*. This correction may not fully accommodate substitution rate variation if the effect is very local or has changed substantially over time.

### Confidence Intervals

Confidence intervals in Figures 3, 4, 5, 6, S2 and S5 were calculated using 1000 bootstrap iterations. Correlation confidence intervals (Figures 7 and S6) were calculated by resampling windows; intervals for the other analyses were calculated by resampling counts of sites in bins, which were assumed to be binomially distributed.

Confidence intervals for maximum likelihood parameter estimates were also calculated by a bootstrap procedure. In each bootstrap iteration, alignment columns were resampled with replacement. As before, columns were binned by their associated exonic and non-exonic *B* values (which differ for each pair of selection coefficients tried), and the local human/dog divergence. Maximum likelihood parameter estimation was done using the binned column counts and a new set of parameter estimates was obtained for each iteration. Confidence intervals for each parameter correspond to the central 90% of the ordered set of estimated values. Confidence intervals for mean autosomal and chromosome X *B* values were calculated using parameter estimates from the same bootstrap iterations. We performed 100 bootstrap iterations, which required approximately six days for the 5-species analysis using a 96-node computer cluster.

## Supporting Information

Figure S1 Mutational events inferred from alignment column types. Solid lines represent a gene tree, and the grey background the species tree (not to scale). Branches are labeled according to the type of alignment column that is generated by a single mutation in that branch. For example, an HCG alignment column, which has the same nucleotides in human, chimpanzee and gorilla, but a different nucleotide that is shared by orangutan and macaque, can be generated by a single mutation that occurred after orangutan speciation but before gorilla speciation. Both HG and CG alignment columns imply a gene tree that differs from the species tree, and a very old human/chimpanzee coalescent that predates gorilla speciation.
(0.42 MB EPS)

Figure S2 Human diversity, interspecies divergence and HG and CG sites are reduced near evolutionarily conserved segments in different genomic regions. We divided the genome into “downstream intergenic regions” (excluding genes and 20 kb upstream of transcription start sites); “upstream intergenic regions” (excluding genes and 20 kb downstream of the polyA site); and intronic sequences (excluding exons, intergenic sequences and first introns). As in Figure 3, ratios were calculated using the 10% of neutral sites which are nearest to and the 50% of neutral sites farthest away from conserved segments, exons or coding sequence (CDS). H/M normalized ratios were also calculated to control for mutation rate variation and sites under purifying selection.
(1.03 MB EPS)

Figure S3 Expected and observed patterns of substitution for a model of background selection fit to 5-species autosomal data. Each plot shows the proportion of sites of a given column type as a function of the estimated strength of background selection, B. The curves and points represent expected and observed column-type proportions from the fitted model: transition substitutions (grey curve, solid black circles), transversion substitutions (blue curve, open blue squares) and conserved sites (red curve, solid red diamonds). For simplicity, the expected curves shown here assume a uniform mutation rate, but during model fitting the mutation rate was allowed to vary regionally. Plots are labelled according to column-types described in Figure S2 (HO+CO columns represent obligate double-substitutions on the H and O or C and O branches). Data is binned by *B* so that each bin contains 10% of the data. Note that the scale of the y-axis differs between plots.
(0.89 MB EPS)

Figure S4 Minor allele frequency distributions for sites near and far from conserved segments. This figure was generated using samples of 32 chromosomes from individuals of African or European descent. Data were obtained from the SeattleSNPs NHLBI Program for Genomic Applications and the NIEHS Environmental Genome Project.
(0.52 MB EPS)

Figure S5 Human/chimpanzee divergence shows a strong dependence on the estimated strength of background selection even after stratifying by local GC content and recombination rate. Human/chimpanzee divergence was calculated from autosomal alignments and binned by B as in Figure 3. Sites were stratified by local GC content (calculated using a 1 Mb sliding window) and recombination rate (from the finescale genetic map of Myers et al.) High, mid and low bins represent the upper quartile, central 50%, and lower quartile bins of the respective distributions. For GC content this corresponds to low≤36%<mid≤43%<high. For recombination rates the following cutoffs were used: low≤0.26 cM/Mb<mid≤0.932 cM/Mb<high. Note that substitution rates have some dependence on GC content; rates are higher in AT-rich regions than in GC-rich regions with the same B value.
(0.30 MB EPS)

Figure S6 Spearman rank correlations of several factors and (A) human/chimpanzee divergence or (B) human nucleotide diversity, at different scales. Non-overlapping autosomal windows of sizes between 212 and 222 bp were used for calculations. Windows with fewer than 10% of sites defined post-filtering were omitted.
(0.42 MB EPS)

Table S1 Spearman rank correlations between conserved segment distances and sequence divergence or diversity. We calculated correlations between distances and neutral divergence/diversity using non-overlapping windows of size 50 kb or 500 kb. Distances were defined as the mean conserved segment distance (or B value) of the unfiltered neutral sites within the window. Only windows where at least 10% of the sites were unfiltered were used. Nucleotide diversity was estimated from Perlegen and HapMap datasets, and divergence was calculated for human/chimp (H/C), human/macaque (H/M) and human/dog (H/D). We also calculated correlations between distance and normalized H/M divergence/diversity.
(0.12 MB DOC)

Table S2 Differences in model log-likelihoods for different selection coefficient distributions. Log-likelihood differences (ΔLL) from the best-fitting distribution are given for the 5-species autosomal (5SA) and human/chimp chromosome X (HCX) data sets. Gamma distributions with three different shape parameters (0.25, 0.75, and 2.0) were tried. The exponential distribution is equivalent to a gamma distribution with shape parameter 1.0. Two models were tried: one in which both exonic and non-exonic conserved segments were considered (B = BexBnex), and one in which only exonic conserved segments were used (B = Bex).
(0.04 MB DOC)

Table S3 Model parameters estimated by maximum likelihood for a simplified model of background selection in which non-exonic conserved segments are ignored. Parameters are as described in Table 1 of the main text.
(0.04 MB DOC)

Table S4 Allele frequency statistics for sites near or far from conserved segments. Single nucleotide polymorphisms (SNPs) were obtained for individuals of European descent (ED) and African descent (AD) from SeattleSNPs EGP and PGA datasets. The following statistics were calculated for sites near or far from conserved segments (as defined in Figure 3). S, number of segregating sites; θW, Watterson's estimator of θ; θT, Tajima's estimator of θ; D, Tajima's D. A p-value is also provided for a two-tailed Kolmogorov-Smirnov test against the null hypothesis that the near and far allele frequency distributions are the same.
(0.07 MB DOC)

Table S5 Nucleotide diversity estimates from the Perlegen dataset. Diversity values (and ratios) are for the 10% or 50% of neutral genomic sites that are nearest-to or farthest-from a conserved segment. Ascertainment-corrected estimates were calculated assuming fixed discovery sample sizes of d = 20 or d = 50 chromosomes.
(0.07 MB DOC)

Table S6 Numbers of column types that result from double substitution events estimated by our background selection model or the method of Patterson et al.
(0.05 MB DOC)

Text S1 Supplementary note on the allele frequency spectrum in sites near and far from conserved segments.
(0.03 MB DOC)
