# Supplementary material for: Widespread Genomic Signatures of Natural Selection in Hominid Evolution
Source: PLoS Genet. 2009 May 8;5(5):e1000471. doi: 10.1371/journal.pgen.1000471 (PMC2669884; doi:10.1371/journal.pgen.1000471)
Supplement: Table S1 — Spearman rank correlations between conserved segment distances and sequence divergence or diversity. We calculated correlations between distances and neutral divergence/diversity using non-overlapping windows of size 50 kb or 500 kb. Distances were defined as the mean conserved segment distance (or B value) of the unfiltered neutral sites within the window. Only windows where at least 10% of the sites were unfiltered were used. Nucleotide diversity was estimated from Perlegen and HapMap datasets, and divergence was calculated for human/chimp (H/C), human/macaque (H/M) and human/dog (H/D). We also calculated correlations between distance and normalized H/M divergence/diversity. (0.12 MB DOC) [file pgen.1000471.s007.doc]

| **Dist. type** | **Win. size (kb)** | **Dataset** | **n win.** | **ρ** | **p-value** | **ρ (H/M norm)** | **p-value (H/M norm)** |
| --- | --- | --- | --- | --- | --- | --- | --- |
| **Exon physical dist** | 50 | Perlegen | 38788 | 0.084 | < 10^-15^ | 0.11 | < 10^-15^ |
|  |  | HapMap | 47325 | 0.073 | < 10^-15^ | 0.11 | < 10^-15^ |
|  |  | H/C | 49455 | 0.30 | < 10^-15^ | 0.12 | < 10^-15^ |
|  |  | H/M | 49455 | 0.45 | < 10^-15^ | NA | < 10^-15^ |
|  |  | H/D | 25527 | 0.32 | < 10^-15^ | -0.16 | < 10^-15^ |
|  |  |  |  |  |  |  |  |
|  | 500 | Perlegen | 4214 | 0.14 | < 10^-15^ | 0.15 | < 10^-15^ |
|  |  | HapMap | 4977 | 0.11 | 8.3×10^-14^ | 0.14 | < 10^-15^ |
|  |  | H/C | 5125 | 0.39 | < 10^-15^ | 0.18 | < 10^-15^ |
|  |  | H/M | 5125 | 0.54 | < 10^-15^ | NA | NA |
|  |  | H/D | 2682 | 0.39 | < 10^-15^ | -0.19 | < 10^-15^ |
|  |  |  |  |  |  |  |  |
| **Exon rec. dist.** | 50 | Perlegen | 38779 | 0.13 | < 10^-15^ | 0.16 | < 10^-15^ |
|  |  | HapMap | 47241 | 0.15 | < 10^-15^ | 0.22 | < 10^-15^ |
|  |  | H/C | 49368 | 0.32 | < 10^-15^ | 0.20 | < 10^-15^ |
|  |  | H/M | 49368 | 0.35 | < 10^-15^ | NA | NA |
|  |  | H/D | 25514 | 0.27 | < 10^-15^ | -0.11 | < 10^-15^ |
|  |  |  |  |  |  |  |  |
|  | 500 | Perlegen | 4213 | 0.22 | < 10^-15^ | 0.23 | < 10^-15^ |
|  |  | HapMap | 4967 | 0.22 | < 10^-15^ | 0.28 | < 10^-15^ |
|  |  | H/C | 5124 | 0.41 | < 10^-15^ | 0.29 | < 10^-15^ |
|  |  | H/M | 5124 | 0.42 | < 10^-15^ | NA | NA |
|  |  | H/D | 2678 | 0.34 | < 10^-15^ | -0.11 | < 10^-15^ |
|  |  |  |  |  |  |  |  |
| ***B*** | 50 | Perlegen | 38788 | 0.23 | < 10^-15^ | 0.18 | < 10^-15^ |
|  |  | HapMap | 47325 | 0.31 | < 10^-15^ | 0.24 | < 10^-15^ |
|  |  | H/C | 49455 | 0.50 | < 10^-15^ | 0.40 | < 10^-15^ |
|  |  | H/M | 49455 | 0.42 | < 10^-15^ | NA | NA |
|  |  | H/D | 25527 | 0.37 | < 10^-15^ | -0.039 | < 10^-15^ |
|  |  |  |  |  |  |  |  |
|  | 500 | Perlegen | 4214 | 0.39 | < 10^-15^ | 0.29 | < 10^-15^ |
|  |  | HapMap | 4977 | 0.47 | < 10^-15^ | 0.32 | < 10^-15^ |
|  |  | H/C | 5125 | 0.63 | < 10^-15^ | 0.56 | < 10^-15^ |
|  |  | H/M | 5125 | 0.51 | < 10^-15^ | NA | NA |
|  |  | H/D | 2682 | 0.49 | < 10^-15^ | -0.015 | 0.43 |
