# Supplementary material for: Widespread Genomic Signatures of Natural Selection in Hominid Evolution
Source: PLoS Genet. 2009 May 8;5(5):e1000471. doi: 10.1371/journal.pgen.1000471 (PMC2669884; doi:10.1371/journal.pgen.1000471)
Supplement: Table S2 — Differences in model log-likelihoods for different selection coefficient distributions. Log-likelihood differences (ΔLL) from the best-fitting distribution are given for the 5-species autosomal (5SA) and human/chimp chromosome X (HCX) data sets. Gamma distributions with three different shape parameters (0.25, 0.75, and 2.0) were tried. The exponential distribution is equivalent to a gamma distribution with shape parameter 1.0. Two models were tried: one in which both exonic and non-exonic conserved segments were considered (B = BexBnex), and one in which only exonic conserved segments were used (B = Bex). (0.04 MB DOC) [file pgen.1000471.s008.doc]

| **Distribution** | **Model** | **ΔLL** |  |
| --- | --- | --- | --- |
|  |  | **5SA** | **HCX** |
| Point | *B*=*B_ex_B_nex_* | -7.0 | 0 |
| Gamma 0.25 |  | -6.2 | -30.9 |
| Gamma 0.75 |  | 0 | -14.8 |
| Exponential |  | -0.3 | -12.9 |
| Gamma 2.0 |  | -6.5 | -9.6 |
| Point | *B*=*B_ex_* | -14.2 | 0 |
| Gamma 0.25 |  | -8.9 | -29.8 |
| Gamma 0.75 |  | -4.3 | -14.8 |
| Exponential |  | -4.6 | -12.6 |
| Gamma 2.0 |  | -11.0 | -5.1 |
