# Supplementary material for: Widespread Genomic Signatures of Natural Selection in Hominid Evolution
Source: PLoS Genet. 2009 May 8;5(5):e1000471. doi: 10.1371/journal.pgen.1000471 (PMC2669884; doi:10.1371/journal.pgen.1000471)
Supplement: Table S3 — Model parameters estimated by maximum likelihood for a simplified model of background selection in which non-exonic conserved segments are ignored. Parameters are as described in Table 1 of the main text. (0.04 MB DOC) [file pgen.1000471.s009.doc]

| **Param** | **Estimates** |  |
| --- | --- | --- |
|  | **5SA** | **HCX** |
| ***μ_I_*** | 7.1×10^-9^ | 6.6×10^-9^ |
| ***μ_V_*** | 1.9×10^-9^ | 1.8×10^-9^ |
| ***λ_I_*** | 2.4 | — |
| ***λ_V_*** | 4.4 | — |
| ***u_ex_*** | 7.5×10^-8^ | 1.6×10^-7^ |
| ***u_nex_*** | — | — |
| ***t_ex_*** | 5.0×10^-3^ | 3.3×10^-3^ |
| ***t_nex_*** | — | — |
| ***T_hc_*** | 2.4×10^5^ (fixed) | 2.4×10^5^ (fixed) |
| ***T_hcg_*** | 1.4×10^5^ | — |
| ***T_hcgo_*** | 4.8×10^5^ | — |
| ***T_hcgom_*** | 7.3×10^5^ | — |
| ***N_hc_*** | 9.5×10^4^ | 4.0×10^4^ |
| ***N_hcg_*** | 5.2×10^4^ | — |
| ***N_hcgo_*** | 8.4×10^4^ | — |
| ***N_hcgom_*** | 6.1×10^4^ | — |
