# Supplementary material for: Widespread Genomic Signatures of Natural Selection in Hominid Evolution
Source: PLoS Genet. 2009 May 8;5(5):e1000471. doi: 10.1371/journal.pgen.1000471 (PMC2669884; doi:10.1371/journal.pgen.1000471)
Supplement: Table S4 — Allele frequency statistics for sites near or far from conserved segments. Single nucleotide polymorphisms (SNPs) were obtained for individuals of European descent (ED) and African descent (AD) from SeattleSNPs EGP and PGA datasets. The following statistics were calculated for sites near or far from conserved segments (as defined in Figure 3). S, number of segregating sites; θW, Watterson's estimator of θ; θT, Tajima's estimator of θ; D, Tajima's D. A p-value is also provided for a two-tailed Kolmogorov-Smirnov test against the null hypothesis that the near and far allele frequency distributions are the same. (0.07 MB DOC) [file pgen.1000471.s010.doc]

| **Pop** | **Cons. type** | **Dist. metric** | **Near sites** |  |  |  | **Far sites** |  |  |  | **KS p-value** |
| --- | --- | --- | --- | --- | --- | --- | --- | --- | --- | --- | --- |
|  |  |  | ***S*** | *** θ_W_*** | ***θ_S_*** | ***D*** | ***S*** | *** θ_W_*** | ***θ_S_*** | ***D*** |  |
| ED | cons. seg. | physical | 1118 | 277.6 | 291.3 | 0.1907 | 1989 | 493.9 | 503.8 | 0.07781 | 0.1904 |
|  |  | deCODE rec. | 1082 | 268.7 | 277.6 | 0.1282 | 2495 | 619.5 | 649.8 | 0.1894 | 0.4873 |
|  |  | Myers rec. | 819 | 203.4 | 201.2 | -0.04153 | 3105 | 771 | 810.4 | 0.1984 | **0.04016** |
|  | exons | physical | 4090 | 1016 | 1046 | 0.1155 | 261 | 64.81 | 61.96 | -0.169 | 0.3942 |
|  |  | deCODE rec. | 3408 | 846.2 | 847.6 | 0.006297 | 384 | 95.35 | 97.49 | 0.08662 | 0.8745 |
|  |  | Myers rec. | 2225 | 552.5 | 540.4 | -0.08487 | 646 | 160.4 | 161.9 | 0.03558 | 0.4572 |
|  | N/A | *B* | 1136 | 282.1 | 258.3 | -0.326 | 2038 | 506.1 | 538.1 | 0.246 | **1.05×10^-7^** |
| AD | cons. seg. | physical | 1818 | 451.4 | 387.7 | -0.5475 | 3248 | 806.5 | 679.9 | -0.6091 | 0.8258 |
|  |  | deCODE rec. | 1729 | 429.3 | 369.1 | -0.5443 | 3980 | 988.3 | 838.9 | -0.5868 | 0.4042 |
|  |  | Myers rec. | 1328 | 329.8 | 264 | -0.7733 | 5076 | 1260 | 1109 | -0.4677 | **5.76×10^-5^** |
|  | exons | physical | 6717 | 1668 | 1430 | -0.5547 | 436 | 108.3 | 87.61 | -0.7364 | 0.4068 |
|  |  | deCODE rec. | 5670 | 1408 | 1190 | -0.6014 | 636 | 157.9 | 131.8 | -0.6394 | 0.9961 |
|  |  | Myers rec. | 3643 | 904.6 | 737.7 | -0.7161 | 1054 | 261.7 | 217.5 | -0.6545 | 0.5367 |
|  | N/A | *B* | 1921 | 477 | 386.4 | -0.7371 | 3151 | 782.4 | 675.8 | -0.529 | **0.01206** |
