# Supplementary material for: Widespread Genomic Signatures of Natural Selection in Hominid Evolution
Source: PLoS Genet. 2009 May 8;5(5):e1000471. doi: 10.1371/journal.pgen.1000471 (PMC2669884; doi:10.1371/journal.pgen.1000471)
Supplement: Table S5 — Nucleotide diversity estimates from the Perlegen dataset. Diversity values (and ratios) are for the 10% or 50% of neutral genomic sites that are nearest-to or farthest-from a conserved segment. Ascertainment-corrected estimates were calculated assuming fixed discovery sample sizes of d = 20 or d = 50 chromosomes. (0.07 MB DOC) [file pgen.1000471.s011.doc]

| **Distance type** |  | **Nucleotide diversity** |  |  |  |  |  | **Nucleotide diversity ratios** |  |  |
| --- | --- | --- | --- | --- | --- | --- | --- | --- | --- | --- |
| **Conserved segment type** | **Distance metric** | **Nearest 10%** |  |  | **Farthest 50%** |  |  | **Near : Far ratio** |  |  |
|  |  | Uncorrected | Corrected (*d*=50) | Corrected (*d*=20) | Uncorrected | Corrected (*d*=50) | Corrected (*d*=20) | Uncorrected | Corrected (*d*=50) | Corrected (*d*=20) |
| Conserved segments | physical | 0.00024 | 0.000244 | 0.000259 | 0.000254 | 0.000258 | 0.000274 | 0.94547 | 0.943448 | 0.943448 |
|  | deCODE recomb. | 0.000225 | 0.000228 | 0.000243 | 0.000263 | 0.000267 | 0.000283 | 0.858222 | 0.856493 | 0.856493 |
|  | Myers *et al.* recomb. | 0.000217 | 0.000221 | 0.000234 | 0.000269 | 0.000274 | 0.000291 | 0.807065 | 0.805995 | 0.805995 |
| Exons | Physical | 0.00021 | 0.000213 | 0.000226 | 0.000281 | 0.000285 | 0.000303 | 0.746762 | 0.74692 | 0.74692 |
|  | deCODE recomb. | 0.000198 | 0.000201 | 0.000214 | 0.000284 | 0.000288 | 0.000306 | 0.697917 | 0.698208 | 0.698208 |
|  | Myers *et al.* recomb. | 0.00019 | 0.000193 | 0.000205 | 0.000285 | 0.000289 | 0.000307 | 0.669154 | 0.66865 | 0.66865 |
| CDS | Physical | 0.000212 | 0.000215 | 0.000228 | 0.00028 | 0.000284 | 0.000302 | 0.756305 | 0.756398 | 0.756398 |
|  | deCODE recomb. | 0.000201 | 0.000204 | 0.000217 | 0.000282 | 0.000287 | 0.000304 | 0.711993 | 0.71239 | 0.71239 |
|  | Myers *et al.* recomb. | 0.000192 | 0.000194 | 0.000206 | 0.000284 | 0.000289 | 0.000306 | 0.674087 | 0.673555 | 0.673555 |
| N/A | *B* | 0.000156 | 0.000158 | 0.000167 | 0.000288 | 0.000292 | 0.00031 | 0.540644 | 0.53922 | 0.53922 |
