# Supplementary material for: Widespread Genomic Signatures of Natural Selection in Hominid Evolution
Source: PLoS Genet. 2009 May 8;5(5):e1000471. doi: 10.1371/journal.pgen.1000471 (PMC2669884; doi:10.1371/journal.pgen.1000471)
Supplement: Table S6 — Numbers of column types that result from double substitution events estimated by our background selection model or the method of Patterson et al. (0.05 MB DOC) [file pgen.1000471.s012.doc]

|  | **This study** |  |  | **Patterson *et al.* 2006** |  |  |
| --- | --- | --- | --- | --- | --- | --- |
| **Column type** | **Number of sites** | **Estimated number of double substitution sites** | **Estimated proportion of double substitution sites** | **Number of sites** | **Estimated number of double substitution sites** | **Estimated proportion of double substitution sites** |
| **H** | 16788 | 32.53942 | 0.00194 | 28504 | 331 | 0.01161 |
| **C** | 15963 | 30.71332 | 0.00192 | 28495 | 331 | 0.01162 |
| **G** | 22922 | 45.68464 | 0.00199 | 38677 | 473 | 0.01223 |
| **HC** | 5025 | 220.4367 | 0.04387 | 8561 | 571 | 0.0667 |
| **HG** | 808 | 232.0741 | 0.28722 | 1302 | 436 | 0.33487 |
| **CG** | 856 | 242.966 | 0.28384 | 1430 | 437 | 0.30559 |
| **HCG** | 25197 | 38.04396 | 0.00151 | 41928 | 2819 | 0.06723 |
| **O** | 48802 | 1054.046 | 0.0216 | 82670 | 1933 | 0.02338 |
| **M** | 145467 | 347.4663 | 0.00239 | 244270 | 1453 | 0.00595 |
| **HO+CO** | 473 | 473 | 1 | 809 | 921 | 1.13844 |
