# Supplementary material for: Widespread Genomic Signatures of Natural Selection in Hominid Evolution
Source: PLoS Genet. 2009 May 8;5(5):e1000471. doi: 10.1371/journal.pgen.1000471 (PMC2669884; doi:10.1371/journal.pgen.1000471)
Supplement: Text S1 — Supplementary note on the allele frequency spectrum in sites near and far from conserved segments. (0.03 MB DOC) [file pgen.1000471.s013.doc]

**Supplementary Text S1**

The frequency of rare alleles has been suggested as a means to distinguish between the effects of hitchhiking and background selection [1,2]. In large populations, background selection should not perturb the allele frequency distribution [2] whereas hitchhiking can result in an excess of rare alleles [3]. In small or moderately sized populations, however, background selection may increase the frequency of rare alleles such that is difficult to distinguish between the two models [2].

To compare the allele frequency distributions of sites that are near or far from conserved segments (Table S4 and Figure S4), we calculated Tajima’s *D* statistic [4]. Under neutrality *D* is expected to be near zero; positive or negative values of *D* indicate an excess of alleles of intermediate or rare frequency, respectively. For both near and far distance bins, *D* falls within the previously observed range for this dataset, and does not have values considered “extreme” (less than -2.0 or greater than 2.0) [5]. When finescale recombination distance from conserved segments or *B* values are used, the differences in the allele frequency spectrum are nominally significant (p < 0.05, two-sided Kolmogorov-Smirnov test). For all significant cases *D* is more negative for “near” sites than “far” sites. This is suggestive of selection, but as the Tajima’s *D* values are not extreme, and the precise effect of background selection on the allele frequency distribution is unclear for moderately-sized populations, we hesitate to draw strong conclusions from this result.

**Methods**

As with other analyses, we used the SeattleSNPs and EGP datasets. To accommodate missing data and ensure a uniform sample size, we resampled 32 alleles without replacement (assuming no linkage) from each polymorphic site in each population. For the ~1% of SNPs that did not have at least 32 chromosomes typed, we resampled with replacement.

**References**

1. Andolfatto P, Przeworski M (2001) Regions of lower crossing over harbor more rare variants in African populations of Drosophila melanogaster. Genetics 158: 657-665.

2. Charlesworth B, Morgan MT, Charlesworth D (1993) The effect of deleterious mutations on neutral molecular variation. Genetics 134: 1289-1303.

3. Braverman JM, Hudson RR, Kaplan NL, Langley CH, Stephan W (1995) The hitchhiking effect on the site frequency spectrum of DNA polymorphisms. Genetics 140: 783-796.

4. Tajima F (1989) Statistical method for testing the neutral mutation hypothesis by DNA polymorphism. Genetics 123: 585-595.

5. Crawford DC, Akey DT, Nickerson DA (2005) The patterns of natural variation in human genes. Annual review of genomics and human genetics 6: 287-312.
